# The effect of a body shape index on physical fitness index is more pronounced in boys than in girls: Evidence from a cross-sectional survey based on Tibetan adolescents aged 13–18 years in high-altitude areas of China

**DOI:** 10.1371/journal.pone.0353705

**Published:** 2026-07-14

**Authors:** Tao Shi, Ke Guan, Chong Liu, Tao Zhang

**Affiliations:** 1 Department of Physical Education and Teaching, Xinjiang Agricultural University, Urumqi, China; 2 School of Physical Education and Sports, Chizhou University, Chizhou, China; 3 Department of Physical Education, Lhasa Teachers’ College, Lhasa, China; Xiangya Hospital Central South University, CHINA

## Abstract

**Background:**

China is an important high-altitude region in the world, mainly inhabited by the Tibetan population. This study was conducted to understand the association between a body shape index (ABSI) and physical fitness index (PFI) among Tibetan adolescents in high-altitude areas of China. This study provides a reference for the improvement of physical fitness and the formulation of public health policies among Tibetan adolescents in high-altitude areas of China.

**Methods:**

Using a stratified whole-group sampling method, 3819 Tibetan adolescents were selected from the high-altitude region of Ganzi, Sichuan, China, and tested on 8 items, including height, weight, waist circumference, grip strength, standing long jump, seated forward bend, 50-meter run, and endurance run (1000m/800m). ABSI and PFI were calculated, and Kruskal-Wallis H was used to compare PFI between groups. Linear regression analysis was used to analyze the correlations that existed between ABSI and PFI.

**Results:**

The ABSI of Chinese Tibetan adolescents aged 13–18 years was (0.09 ± 0.01). Among them, ABSI was (0.09 ± 0.01) for boys and (0.09 ± 0.01) for girls, with no significant difference in comparison (*t* = 1.594, *P* > 0.05). PFI [M(P_25_,P_75_)] for Tibetan adolescents aged 13−18 years was −0.37 (−2.31,1.48), among them, PFI for boys was – 0.24 (−2.41,1.63) and the girl’s PFI was −0.46(−2.20,1.35), with no significant difference in comparison (*Z* value = −0.878, *P* > 0.05). Overall, the comparison of PFI between the different ABSI groups (A, B, C, and D) was statistically significant (*P* < 0.01) in all age groups except for the 17-year-old age group where there was no significant difference (*P* > 0.05).

**Conclusion:**

The ABSI level of Tibetan adolescents in high-altitude areas in China is low and negatively correlated with PFI. Compared with girls, the effect of ABSI on PFI was more obvious in boys. It is suggested that in the future, Tibetan adolescents in high-altitude areas should control the occurrence of waist circumference and overweight obesity and increase physical exercise to promote the physical fitness level of Tibetan adolescents.

## 1. Introduction

A body shape index (ABSI) is an index that effectively reflects the ratio between body waist circumference and overall obesity of adolescents. ABSI was developed by foreign scholars Krakauer et al and has gradually been widely recognized and applied by domestic and foreign scholars [1,2]. Studies have shown that there is a close correlation between ABSI and several body indices [3–7]. Also, ABSI is more accurate in predicting various types of chronic cardiovascular diseases, diabetes, and all-cause mortality in adolescents compared with BMI, waist circumference, and waist-height ratio [8–14]. However, some scholars have shown that there is no significant association between ABSI and body blood pressure, and the reason for this may be related to some differences in the population investigated in different studies [15].

Tibetans, as one of China’s ethnic minorities, live mainly in the Qinghai-Tibetan plateau region and the Ganzi region of Sichuan, which is known as the “roof of the world” in China. These high-altitude regions have low oxygen, high ultraviolet rays, and infertile land all year round, resulting in the development of special plateau-adapted physical characteristics in the youth of these regions [16–18]. Studies have shown that Tibetan children and adolescents in high-altitude areas of China have lower levels of health fitness and higher detection rates of malnutrition [19,20]. The study also showed that the lower limb muscle strength of Chinese Tibetan children and adolescents needs to be improved and the effects of age, gender, and obesity factors should be considered [21–23].

The physical fitness index (PFI), as a comprehensive index reflecting the physical fitness level of adolescents, has been widely adopted by scholars at home and abroad and can reflect the physical fitness level of adolescents comprehensively [24]. Previous studies have mainly focused on the relationship between BMI and a physical fitness index, such as BMI and cardiorespiratory endurance and muscular strength [25,26]. There are fewer studies on the relationship between ABSI and PFI composite indexes in adolescents at high altitudes.

To the best of our knowledge, no current studies on the relationship between ABSI and PFI in Tibetan adolescents at high altitudes in China have been found. Therefore, we tested physical fitness and body morphology in 3819 Tibetan adolescents at high altitudes in China. To analyze the association between ABSI and PFI in Tibetan adolescents at high altitudes in China. To provide a reference and basis for the improvement of the physical fitness level of Tibetan adolescents in high-altitude areas of China and for the formulation of public health and education policies by government departments.

## 2. Materials and methods

### 2.1 Subjects

**Fig 1:**
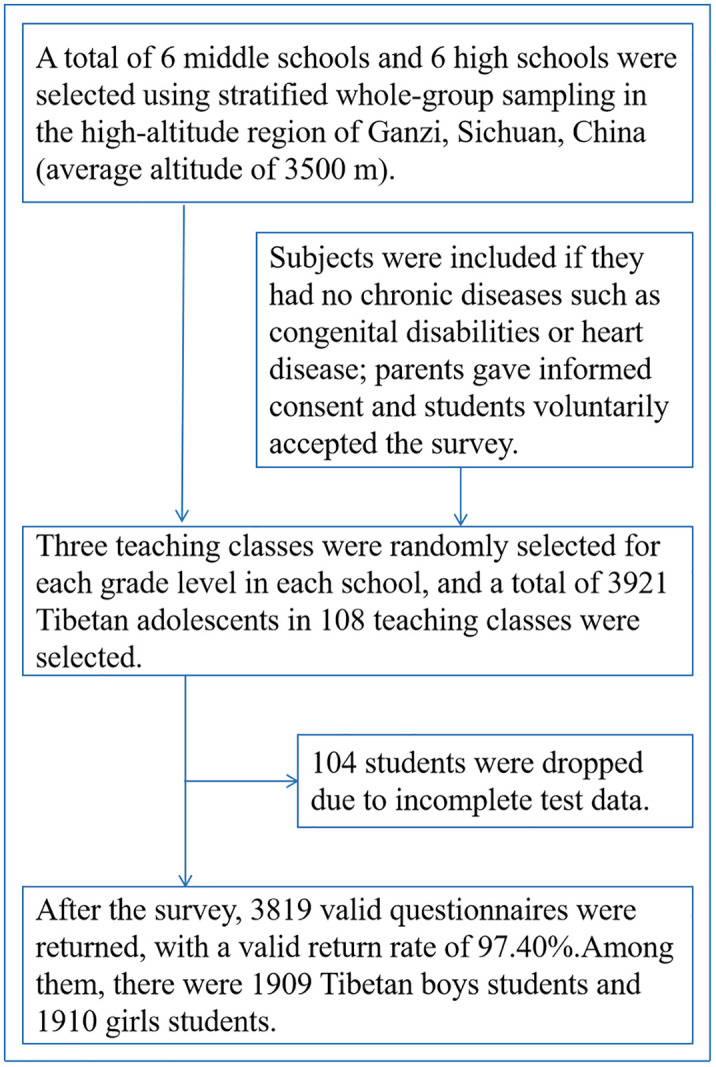
Sampling process of Tibetan adolescents in high-altitude areas of China.

Participants in this study will be evaluated from September to October 2024. Based on the sampling methods used in the China National Student Physical Fitness Survey [27]. A total of 6 middle schools and 6 high schools were selected using stratified whole-group sampling in the high-altitude region of Ganzi, Sichuan, China (average altitude of 3500 m). Three teaching classes were randomly selected for each grade level in each school, and a total of 3921 Tibetan adolescents in 108 teaching classes were selected. Participants were selected using a random sampling method. Subjects were included if they had no chronic diseases such as congenital disabilities or heart disease; parents gave informed consent and students voluntarily accepted the survey. In this study, chronic diseases refer to a category of noncommunicable diseases characterized by a long course of illness, gradual onset, complex etiology, and difficulty in achieving a complete cure, requiring long-term monitoring and comprehensive management; examples include hypertension, coronary heart disease, and diabetes. 104 students were dropped due to incomplete test data. These primarily include key demographic trends, such as age and gender; and incomplete information due to damaged questionnaires. After the survey, 3819 valid questionnaires were returned, with a valid return rate of 97.40%. The specific sampling process of the subjects in this study is shown in Fig 1.

The study was conducted according to the World Medical Association Declaration of Helsinki. The study was granted with informed consent from the students and parents and written informed consent was signed. The study was approved by the Ethics Committee of Xinjiang Agricultural University (202445784).

### 2.2 Test indicators

#### 2.2.1 A body shape index (ABSI).

The formula of ABSI is waist circumference/(body mass index 2/3*height 1/2) [1], and the formula of body mass index (BMI) is weight (kg)/height (m)^2^. According to the calculation formula, the tests of height, weight, and waist circumference are required. The tests were performed according to the methods and instruments required by the Chinese National Student Physical Health Survey, with height and waist circumference accurate to 0.1 cm and weight accurate to 0.1 kg [27].

#### 2.2.2 Physical Fitness Index (PFI).

The physical fitness test items in our study were: grip strength and standing long jump, which reflect muscle strength; seated forward bend, which reflects flexibility quality; 50-meter run, which reflects speed quality; and 1000-meter/800-meter run, which reflects endurance quality [27]. The test methods were conducted according to the methods and instruments required by the China National Student Physical Fitness Survey [27]. After the test, the mean value of each gender and age category of each item in the 2014 China National Student Physical Fitness Survey was used as a reference for the calculation of the Z-score, which was calculated as (actual test value – national average reference value in China)/China national standard deviation [28].

PFI was the Z _grip strength_ + Z _Sit and reach_ + Z _standing long jump_ – Z _50-m race_ – Z _endurance run (1000 m/800 m)_.

Since the 50m and 1000m/800m runs are speed events, the shorter the time, the better the performance of the event, so the subtraction method is used [27]. The higher the PFI value, the better the physical fitness level of the subjects [27].

### 2.3 Quality control

The physical fitness items tested in our study were administered by highly trained teachers who served as test staff. The tests were administered by a fixed and dedicated staff, and the tests were conducted in strict accordance with the training requirements and procedures. The test was conducted after the calibration of the instruments before the daily test. Test scores were filled in by the testers on the students’ test cards [27].

### 2.4 Statistical analysis

Since the PFI was non-normally distributed data, the median P_25_ and P_75_ were used for representation. Based on the quartiles, the ABSI of Tibetan adolescents in high-altitude areas of China was divided into four groups: ABSI < P_25_ group, ABSI P_25-50_ group, ABSI P_51-75_ group, and ABSI > P_75_ group. The comparison of PFI between different ABSI groups was performed using Kruskal-Wallis H. Comparison of ABSI between different genders was performed using a t-test. Comparisons of PFI between different genders were performed using the Mann-Whitney U test.

The relationship between ABSI and PFI was stratified by gender, and linear regression analysis was performed using PFI as the dependent variable and ABSI as the independent variable among Tibetan adolescents in high-altitude areas of China. The specific equation is:


$$\begin{array}{c} Y=aX^{2}+bX+c\;(Y,\;PFI;\;X,\;ABSI) \end{array}$$

Where a (nonlinear coefficient), b (linear coefficient), and c (intercept) are constants. Curvilinear regression analysis was performed with Y as the dependent variable and X as the independent variable. A two-sided test level of *α* = 0.05 was used. Data were processed and analyzed using SPSS 25.0 (IBM Inc., Armonk, NY, USA) software.

## 3. Results

After the survey, 3819 valid questionnaires were returned, among them, there were 1908 Tibetan boys students and 1909 girls students. The average age of Tibetan boys students was (15.51 ± 1.69) years old and Tibetan girls students were (15.54 ± 1.69) years old.

According to Table 1, The ABSI of Chinese Tibetan adolescents aged 13–18 years was (0.09 ± 0.01). Among them, ABSI was (0.09 ± 0.01) for boys and (0.09 ± 0.01) for girls, with no significant difference in comparison (*t* = 1.594, *P* = 0.111). PFI [M(P_25_, P_75_)] for Tibetan adolescents aged 13−18 years was −0.37 (−2.31,1.48), among them, PFI for boys was – 0.24 (−2.41,1.63) and the girl’s PFI was −0.46(−2.20,1.35), with no significant difference in comparison (*Z* value = −0.878, *P* > 0.05).

**Table 1:** Basic characteristics of participants among Tibetan adolescents aged 13-18 years in high-altitude areas of China[M ± SD; M(*P*_25_, *P*_75_)].

Type	Boys	Girls	Total	*T/H-*value	*P-*value
Number	1909	1910	3819		
Age(years)	15.51 ± 1.69	15.54 ± 1.69	15.52 ± 1.69	−0.521	0.958
Height(cm)	164.60 ± 9.30	156.41 ± 5.86	160.50 ± 8.78	32.536	<0.001
Weight(kg)	53.68 ± 10.53	51.00 ± 8.19	52.34 ± 9.53	8.775	<0.001
BMI(kg/m^2^)	19.67 ± 2.79	20.80 ± 2.84	20.24 ± 2.87	−12.321	0.005
Waist Circumference(cm)	69.28 ± 7.44	69.28 ± 7.08	69.28 ± 7.26	0.030	0.976
Grip strength(kg)	35.97 ± 9.78	25.57 ± 5.59	30.77 ± 9.51	40.312	<0.001
Standing long jump(cm)	203.37 ± 28.36	152.97 ± 19.46	178.16 ± 35.02	64.046	<0.001
Sit and reach(cm)	8.81 ± 6.31	10.81 ± 5.97	9.81 ± 6.22	−10.057	<0.001
50-m race(s)	8.05 ± 1.36	9.65 ± 0.97	8.85 ± 1.42	−41.887	<0.001
1000/800-meter race(s)	269.56 ± 38.84	255.87 ± 30.08	——	——	
A body shape index	0.09 ± 0.01	0.09 ± 0.01	0.09 ± 0.01	1.594	0.111
Physical fitness index	−0.24(−2.41,1.63)	−0.46(−2.20,1.35)	−0.37(−2.31,1.48)	−0.878	0.380

The BMI, waist, circumference, grip strength, standing long jump, sit and reach, 50-m race, 1000/800-meter race scores of Tibetan adolescents aged 13–18 years old in high altitude areas of China were (20.24 ± 2.87) kg/m^2^, (69.28 ± 7.26)cm, (30.77 ± 9.51)kg, (178.16 ± 35.02)cm, (9.81 ± 6.22)cm, (8.85 ± 1.42)s, (269.56 ± 38.84)s, (255.87 ± 30.08)s, respectively.

Table 2 shows that the comparison of PFI values among Tibetan adolescents in high-altitude areas of China was statistically significant in the age groups of 13, 17, and 18 years old (*P* < 0.05).

**Table 2:** Comparison of PFI values among Tibetan adolescents aged 13-18 years in high-altitude regions of China[M(*P*_25_,*P*_75_)].

Age(years)	Boys	Girls	Total	*H-*value	*P-*value
13	−1.41(−3.91,0.94)	−0.42(−2.02,1.23)	−0.76(−3.24,1.14)	−3.587	<0.001
14	−0.75(−2.38,1.30)	−0.40(−1.84,1.10)	−0.53(−2.20,1.18)	−1.078	0.281
15	−1.27(−3.20,0.81)	−1.21(−3.08,0.57)	−1.22(−3.13,0.70)	−0.032	0.974
16	0.48(−1.70,2.01)	−0.04(−2.06,1.85)	0.22(−1.85,1.96)	−0.838	0.402
17	0.36(−1.37,2.02)	−0.31(−2.03,1.39)	0.06(−1.67,1.80)	−3.143	0.002
18	0.30(−1.54,1.90)	−0.35(−2.06,1.75)	0.05(−1.83,1.81)	−2.142	0.032
13-18	−0.24(−2.41,1.63)	−0.46(−2.20,1.35)	−0.37(−2.31,1.48)	−0.878	0.380

Table 3 shows the comparison of PFI among different ABSI groups of Tibetan adolescents aged 13–18 years in high-altitude areas of China. Overall, the comparison of PFI between the different ABSI groups (A, B, C, and D) was statistically significant (*P* < 0.01) in all age groups except for the 17-year-old age group where there was no significant difference (*P* > 0.05). Statistical significance (*P* < 0.01) was also found in all age groups for boys, except for the 17-year-old age group where there was no significant difference (*P* > 0.05). Statistical significance was found in girls only in the age group of 13 and 18 years (*P* < 0.01). Supplementary table 1 shows Post hoc pairwise comparisons of PFI scores among different ABSI groups in Tibetan adolescents aged 13–18 in high-altitude regions of China.

**Table 3:** Comparison of PFI among different ABSI groups among Tibetan adolescents aged 13-18 years in high altitude areas of China [M(*P*_25_,*P*_75_)].

Gender/ Age(yr)	ABSI<25th Percentile(A)		25th≤ABSI<50th Percentile(B)		50th≤ABSI<75th Percentile(C)		ABSI≥75th Percentile(D)		*H-*value	*P-*value
	N	M (*P*25, *P*75)	N	M (*P*25, *P*75)	N	M (*P*25, *P*75)	N	M (*P*25, *P*75)		
Boys										
13	18	0.48(−1.96,3.91)	46	0.60(−1.20,3.01)	67	0.27(−2.31,2.46)	182	−2.74(−5.18,-0.19)	42.391	<0.01
14	30	0.66(−1.89,3.35)	58	0.20(−2.02,2.12)	86	0.06(−1.64,1.40)	139	−1.36(−3.44,0.06)	19.766	<0.01
15	41	0.45(−2.10,3.46)	82	−0.33(−1.96,1.50)	89	−1.60(−3.05,0.70)	102	−2.74(−5.00,-0.56)	27.941	<0.01
16	131	1.01(−0.89,2.81)	87	0.22(−1.53,2.09)	72	0.02(−1.88,1.21)	49	−0.97(−3.68,0.81)	16.495	<0.01
17	147	0.88(−0.79,2.16)	85	0.06(−1.92,1.81)	63	0.30(−1.47,1.83)	28	−0.26(−1.51,1.24)	6.011	0.111
18	144	0.87(−0.99,2.21)	72	−0.04(−1.45,1.74)	71	−0.21(−1.91,1.56)	20	−0.60(−3.34,1.41)	9.760	<0.05
Girls										
13	37	−0.02(−1.62,1.73)	61	0.27(−1.17,1.69)	77	−0.01(−1.94,1.77)	124	−1.17(−2.88,0.22)	22.815	<0.01
14	65	0.30(−1.49,2.13)	89	−0.17(−1.79,1.15)	95	−0.67(−1.71,0.79)	63	−0.88(−3.00,0.73)	6.941	0.074
15	67	−1.00(−2.49,0.86)	91	−1.57(−3.32,1.37)	104	−1.36(−3.23,0.28)	70	−1.17(−2.98,0.08)	1.320	0.724
16	90	0.40(−1.79,2.54)	91	0.14(−1.82,1.85)	73	−0.12(−2.22,2.16)	66	−0.65(−2.21,1.20)	4.369	0.224
17	93	−0.13(−1.60,2.06)	106	−0.22(−2.01,1.22)	76	−0.52(−2.27,1.12)	54	−0.51(−2.13,1.17)	0.172	0.982
18	90	0.34(−1.31,2.11)	92	0.55(−1.80,1.90)	85	−0.81(−2.15,0.98)	51	−1.44(−2.97,0.30)	18.013	<0.01
Total										
13	55	0.25(−1.73,2.35)	107	0.36(−1.14,2.14)	144	0.16(−2.11,1.96)	306	−1.85(−4.24,-0.04)	74.708	<0.01
14	95	0.44(−1.62,2.60)	147	−0.05(−1.79,1.88)	181	−0.20(−1.65,1.12)	202	−1.20(−3.39,0.23)	34.894	<0.01
15	108	−0.65(−2.48,1.69)	173	−0.57(−2.52,1.41)	193	−1.56(−3.07,0.49)	172	−2.07(−4.21,-0.36)	30.970	<0.01
16	221	0.85(−1.32,2.77)	178	0.18(−1.57,1.86)	145	−0.03(−2.10,1.33)	115	−0.80(−2.69,0.97)	28.938	<0.01
17	240	0.33(−1.21,2.07)	191	−0.14(−1.99,1.50)	139	−0.06(−2.04,1.35)	82	−0.39(−1.87,1.17)	6.593	0.086
18	234	0.59(−1.08,2.15)	164	0.35(−1.57,1.87)	156	−0.53(−2.13,1.38)	71	−1.23(−2.97,0.69)	30.194	<0.01

Note: ^a^ ≤ 0.05, ^b^ ≤0.01, ^c^ ≤0.001.

**Fig 2:**
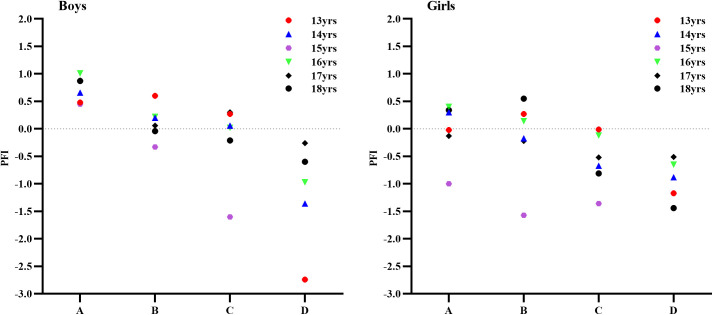
Trends in PFI Values of Tibetan Adolescents Aged 13-18 Years in High Altitude Areas of China by ABSI Grade. Note: ABSI<25th Percentile(A), 25th≤ABSI<50th Percentile(B), 50th≤ABSI<75th Percentile(C), ABSI≥75th Percentile(D).

Fig 2 shows the trend of PFI values for different ABSI levels among Tibetan adolescents aged 13–18 years in high-altitude areas of China. With the increase of ABSI, the overall PFI showed a decreasing trend.

With PFI of Tibetan adolescents as the dependent variable and ABSI as the independent variable, a quadratic term curve regression analysis was performed to derive the regression equations. Respectively:


$$\begin{array}{c} Boys:\;Y=-{\text{1}\text{4}\text{6}\text{8}.\text{3}\text{8}\text{5}X}^{\text{2}}+\text{1}\text{6}\text{9}.\text{6}\text{9}\text{8}X-\text{3}.\text{8}\text{8}\text{5}\to R^{\text{2}}\;=\text{0}.\text{1}\text{4}\text{8} \end{array}$$

$$\begin{array}{c} Girls:\;Y={\text{9}\text{3}.\text{2}\text{8}\text{4}X}^{\text{2}}-\text{4}\text{7}.\text{6}\text{7}\text{9}X+\text{2}.\text{9}\text{6}\text{9}\;\to \;R^{\text{2}}\;=\text{0}.\text{0}\text{1}\text{4} \end{array}$$

$$\begin{array}{c} Total:\;Y={-\text{6}\text{7}.\text{8}\text{4}\text{8}X}^{\text{2}}-\text{5}\text{3}.\text{7}\text{8}\text{3}X+\text{4}.\text{7}\text{4}\text{6}\to R^{\text{2}}\;=\text{0}.\text{0}\text{6}\text{6} \end{array}$$

Y, PFI; X, ABSI\)

**Fig 3:**
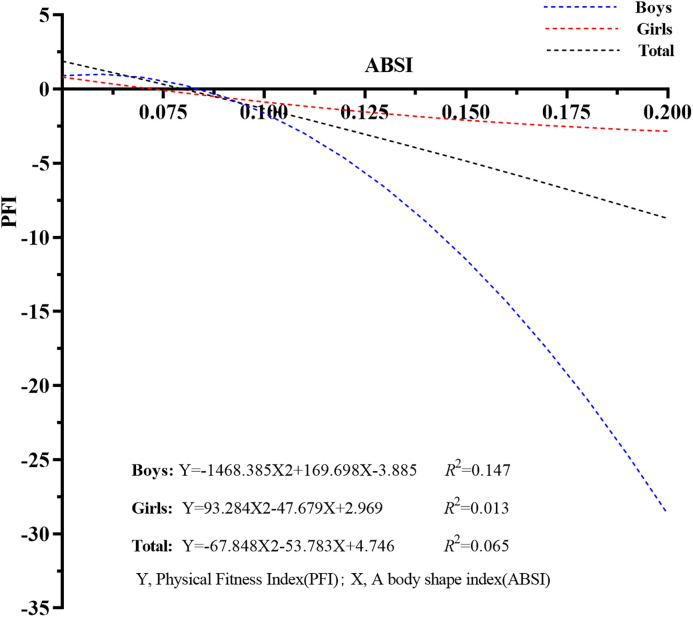
Trends of ABSI and PFI in Tibetan adolescents aged 13-18 years in high-altitude areas of China.

Fig 3 shows the trend of ABSI and PFI among Tibetan adolescents aged 13–18 years in high-altitude areas of China. It can be seen that the level of PFI among Tibetan adolescents showed a decreasing trend with the increase of ABSI. Compared with girls, the effect of ABSI on PFI was more obvious in boys.

## 4. Discussion

Our study showed that the ABSI of Tibetan adolescents in high-altitude areas of China was (0.09 ± 0.01). This result is low compared to the findings of ABSI among Malaysian adolescents (0.1389 ± 0.0074) [29]. Since there are no findings for ABSI among adolescents in high-altitude areas in China, cross-sectional comparisons are not possible. However, the results of our study also indicate to some extent that the level of ABSI among Tibetan adolescents in high-altitude areas in China is at a low level compared to foreign studies. The reasons for this result: on the one hand, the overall low level of economic development in the high-altitude region of Ganzi, Sichuan, China, where our study was investigated, is an important reason for the lower ABSI levels among Tibetan adolescents in this region. Relevant studies have confirmed that there is a positive correlation between ABSI and the level of regional economic development, which can better explain our findings [13]. On the other hand, the influence of factors such as dietary habits and lifestyle of Chinese Tibetan adolescents led to the thin body size of Tibetan adolescents in our study, which resulted in lower ABSI levels. Some findings show that the proportion of wasted or malnourished Chinese Tibetan adolescents is higher compared to the eastern plains, which is another important reason for the lower ABSI levels in our study [30].

Regarding PFI in each ABSI group, our study showed that overall PFI levels were lower in Chinese Tibetan adolescents in the ABSI value > P_75_ group. It indicates that either physical obesity or large waist circumference values lead to lower PFI levels, i.e., affect physical fitness levels. Studies have shown that there is a correlation between BMI and PFI, with adolescents in the higher or lower BMI groups having lower PFI levels [31]. It has also been shown that heavier or obese adolescents need to overcome greater body weight resistance during tests such as endurance running, resulting in lower fitness levels, which is an important reason for the lower fitness levels in the higher ABSI group [32]. This result also suggests that we should pay special attention to the improvement of the physical fitness level of overweight or obese Tibetan adolescents in high-altitude areas in the future.

The correlation analysis showed that there was a negative association between ABSI and PFI among Tibetan adolescents at high altitudes in China, i.e., higher ABSI levels were associated with relatively lower PFI levels. This result also suggests that we should effectively control the increase of waist circumference or BMI level in Tibetan adolescents in high altitude areas of China in the future to reduce the proportion of overweight and obesity to better promote the increase of physical fitness level. Studies have also shown that PFI levels of overweight obese Chinese children and adolescents are significantly lower than those of normal-weight adolescents, which also indicates the importance of maintaining normal body size to promote and improve physical fitness levels [33,34]. Other studies on Chinese Xinjiang adolescents also showed that there is a negative association between PFI levels and overweight obesity and that BMI should be effectively controlled to improve physical fitness levels and to guarantee the improvement of physical fitness levels among adolescents in border areas [35,36].

Our study has certain advantages. First, to our knowledge, this is the first study on the association between body size and physical fitness in Tibetan adolescents in high-altitude areas of China. Second, both ABSI and PFI are comprehensive indicators, which can provide a comprehensive picture of the subjects’ body size and physical fitness levels. However, there are some limitations to our study. First, the study was a cross-sectional regression study, and it was not possible to understand the causal associations between them. Second, our study was conducted on Tibetan adolescents in the high-altitude region of Ganzi, Sichuan, China, but not in a broader high-altitude region, such as Tibet, China. These deficiencies need to be remedied in future studies. In addition, future studies should evaluate the participants’ post-test status, including their recovery following the physical fitness tests.

## 5. Conclusion

The level of ABSI in Tibetan adolescents at high altitudes in China is low and has a negative association with the level of PFI. Compared with girls, the effect of ABSI on PFI was more obvious in boys. In the future, the incidence of waist circumference and overweight obesity among Tibetan adolescents in high-altitude areas of China should be controlled to promote the improvement of PFI levels. Of course, besides effectively controlling the occurrence of obesity, it should also effectively prevent the occurrence of malnutrition or wasting ratio among Tibetan adolescents in high-altitude areas, which also has an important role and significance in promoting and improving their physical fitness. Our findings also suggest that in the future, special attention should be paid to the control of ABSI in Tibetan boys at high altitudes to keep it in a reasonable range to prevent the decrease of PFI.

## Supporting information

S1 Table Post hoc pairwise comparisons of PFI scores among different ABSI groups in Tibetan adolescents aged 13–18 in high-altitude regions of China [M(*P*_25_,*P*_75_)]. Note: ^a^ < 0.05, ^b^ < 0.01, ^c^<0.001. ^#^ Comparison of ABSI between groups, *H*-value and *P.* ABSI<25th Percentile(A), 25th≤ABSI<50th Percentile(B), 50th≤ABSI<75th Percentile(C), ABSI≥75th Percentile(D).
(DOCX)
