# Supplementary material for: The effect of a body shape index on physical fitness index is more pronounced in boys than in girls: Evidence from a cross-sectional survey based on Tibetan adolescents aged 13–18 years in high-altitude areas of China
Source: PLoS One. 2026 Jul 14;21(7):e0353705. doi: 10.1371/journal.pone.0353705 (PMC13367736; doi:10.1371/journal.pone.0353705)
Supplement: S1 Table — Note: a < 0.05, b < 0.01, c<0.001. # Comparison of ABSI between groups, H-value and P. ABSI<25th Percentile(A), 25th≤ABSI<50th Percentile(B), 50th≤ABSI<75th Percentile(C), ABSI≥75th Percentile(D). (DOCX) [file pone.0353705.s001.docx]

**Supplementary table** **1** Post hoc pairwise comparisons of PFI scores among different ABSI groups in Tibetan adolescents aged 13 to 18 in high-altitude regions of China [M(*P*_25_,*P*_75_)]

| Gender/  Age(yr) | *H-*value/*P* ^#^ | | | | | |
| --- | --- | --- | --- | --- | --- | --- |
|  | A/B | A/C | A/D | B/C | B/D | C/D |
| Boys |  |  |  |  |  |  |
| 13 | -0.31 | -0.60 | -3.26^b^ | -1.17 | -5.15^c^ | -4.89^c^ |
| 14 | -0.71 | -1.17 | -3.32^b^ | -0.72 | -3.70^c^ | -3.74^c^ |
| 15 | -1.30 | -3.00^b^ | -5.01^c^ | -2.65^b^ | -5.66^c^ | -3.23^b^ |
| 16 | -2.39^a^ | -3.52^c^ | -4.92^c^ | -1.20 | -3.13^b^ | -2.19^a^ |
| 17 | -1.54 | -0.99 | -1.46 | -0.45 | -0.13 | -0.75 |
| 18 | -1.47 | -2.42^a^ | -2.19^a^ | -0.95 | -1.35 | -0.71 |
| Girls |  |  |  |  |  |  |
| 13 | -0.25 | -0.27 | -2.74^b^ | -0.75 | -4.07^c^ | -3.18^b^ |
| 14 | -0.86 | -2.16^a^ | -2.87^b^ | -0.99 | -2.11^a^ | -1.36 |
| 15 | -0.01 | -0.77 | -0.81 | -0.51 | -0.70 | -0.10 |
| 16 | -0.60 | -1.13 | -1.89 | -0.56 | -1.62 | -0.67 |
| 17 | -0.40 | -0.89 | -0.85 | -0.62 | -0.39 | -0.10 |
| 18 | -0.48 | -2.66^a^ | -3.81^b^ | -2.30^a^ | -3.30^b^ | -1.42 |
| Total |  |  |  |  |  |  |
| 13 | -0.45 | -0.55 | -4.67^c^ | -1.24 | -7.01^c^ | -6.15^c^ |
| 14 | -0.92 | -2.19^a^ | -4.83^c^ | -1.17 | -4.54^c^ | -3.87^c^ |
| 15 | -0.50 | -2.40^a^ | -4.52^c^ | -2.23^a^ | -4.82^c^ | -2.69^b^ |
| 16 | -2.29 | -3.36^b^ | -4.99^c^ | -1.27 | -3.19^c^ | -1.90 |
| 17 | -1.86 | -1.75 | -2.16a | -0.07 | -0.50 | -0.56 |
| 18 | -1.42 | -3.82^c^ | -4.69^c^ | -2.36^a^ | -3.63^c^ | -1.84 |

Note： ^a^ ＜0.05，^b^ ＜0.01，^c^ ＜0.001. ^#^ Comparison of ABSI between groups, *H*-value and *P.* ABSI＜25th Percentile(A), 25th≤ABSI＜50th Percentile(B), 50th≤ABSI＜75th Percentile(C), ABSI≥75th Percentile(D).
