# Supplementary material for: The effect of a body shape index on physical fitness index is more pronounced in boys than in girls: Evidence from a cross-sectional survey based on Tibetan adolescents aged 13–18 years in high-altitude areas of China
Source: PLoS One. 2026 Jul 14;21(7):e0353705. doi: 10.1371/journal.pone.0353705 (PMC13367736; doi:10.1371/journal.pone.0353705)
Supplement: Attachment — Submitted filename: Response to Reviewers.docx [file pone.0353705.s003.docx]

Journal Requirements:

When submitting your revision, we need you to address these additional requirements.

1. Please ensure that your manuscript meets PLOS ONE's style requirements, including those for file naming. The PLOS ONE style templates can be found at

https://journals.plos.org/plosone/s/file?id=wjVg/PLOSOne_formatting_sample_main_body.pdf and

https://journals.plos.org/plosone/s/file?id=ba62/PLOSOne_formatting_sample_title_authors_affiliations.pdf

2. Thank you for stating the following in the Acknowledgments Section of your manuscript:

“This study was supported by the Xinjiang Social Science Foundation Program (22BTY083).”

We note that you have provided funding information that is not currently declared in your Funding Statement. However, funding information should not appear in the Acknowledgments section or other areas of your manuscript. We will only publish funding information present in the Funding Statement section of the online submission form.

Please remove any funding-related text from the manuscript and let us know how you would like to update your Funding Statement. Currently, your Funding Statement reads as follows:

“This study was supported by the Xinjiang Social Science Foundation Program (22BTY083)”

Please include your amended statements within your cover letter; we will change the online submission form on your behalf.

Revision response: We appreciate the reviewers’ valuable suggestions. We have removed the funding information from the manuscript and added the grant details in the submission system.

3. We note that the grant information you provided in the ‘Funding Information’ and ‘Financial Disclosure’ sections do not match.

When you resubmit, please ensure that you provide the correct grant numbers for the awards you received for your study in the ‘Funding Information’ section.

Revised response: We appreciate the reviewers’ valuable suggestions and have made the requested revisions.

4. Thank you for stating the following financial disclosure:

“This study was supported by the Xinjiang Social Science Foundation Program (22BTY083)”

Please state what role the funders took in the study.  If the funders had no role, please state: "The funders had no role in study design, data collection and analysis, decision to publish, or preparation of the manuscript."

If this statement is not correct you must amend it as needed.

Please include this amended Role of Funder statement in your cover letter; we will change the online submission form on your behalf.

Revised response: We appreciate the reviewers’ valuable suggestions and have made the requested revisions.

5. PLOS requires an ORCID iD for the corresponding author in Editorial Manager on papers submitted after December 6th, 2016. Please ensure that you have an ORCID iD and that it is validated in Editorial Manager. To do this, go to ‘Update my Information’ (in the upper left-hand corner of the main menu), and click on the Fetch/Validate link next to the ORCID field. This will take you to the ORCID site and allow you to create a new iD or authenticate a pre-existing iD in Editorial Manager.

Revised response: We appreciate the reviewers’ valuable suggestions and have made the requested revisions.

6. Your ethics statement should only appear in the Methods section of your manuscript. If your ethics statement is written in any section besides the Methods, please delete it from any other section.

Revised response: We appreciate the reviewers’ valuable suggestions and have made the requested revisions.

7. Please remove your figures from within your manuscript file, leaving only the individual TIFF/EPS image files, uploaded separately. These will be automatically included in the reviewers’ PDF.

Revised response: We appreciate the reviewers’ valuable suggestions and have made the requested revisions.

8. We are unable to open your Supporting Information file “Supporting Information.zip”. Please kindly revise as necessary and re-upload.

Revised response: We appreciate the reviewers’ valuable suggestions and have made the requested revisions.

9. If the reviewer comments include a recommendation to cite specific previously published works, please review and evaluate these publications to determine whether they are relevant and should be cited. There is no requirement to cite these works unless the editor has indicated otherwise. 
Revised response: We appreciate the reviewers’ valuable suggestions and have made the requested revisions.

Additional Editor Comments:

1.The manuscript provides different sample sizes in different sections. The abstract and methods state 3819 valid participants, whereas the introduction states 3817 adolescents were tested. In the Results section, the text reports 3817 valid questionnaires, including 1908 boys and 1909 girls, while Table 1 reports 1909 boys, 1910 girls, and 3819 total.
Revised response: We appreciate the reviewers’ valuable suggestions and have made the requested revisions. The number of participants was 3,819.

2. Table 1 contains multiple apparent statistical errors. For example, the text states that ABSI did not differ significantly by sex, with t = 1.594, P > 0.05, but Table 1 reports P < 0.001 for ABSI. Other examples also require verification. Waist circumference is reported as 69.28 ± 7.44 cm in boys and 69.28 ± 7.08 cm in girls, with t = 0.030, but the P-value is reported as 0.002, which is inconsistent. Sit-and-reach has t = -10.057, but P = 0.075, which is also implausible.
Revised response: We appreciate the reviewers’ valuable suggestions and have made the requested revisions. I apologize for the careless mistake; an error occurred while entering the data.

3. The authors state that they adjusted for age and BMI and then performed quadratic regression with PFI as the dependent variable and ABSI as the independent variable. However, the reported equations include only ABSI terms and an intercept. If age and BMI were adjusted, the regression equations should include these covariates, or the authors should clearly explain how adjustment was performed. The manuscript also does not provide regression coefficients, standard errors, 95% confidence intervals, P-values, or model diagnostics. Without these, the conclusion that the effect of ABSI on PFI is stronger in boys than girls are not adequately supported.
Revised response: We appreciate the reviewers’ valuable suggestions and have made the requested revisions. The *R*² values for the equations are reported in the manuscript. The analysis was stratified by sex.

4. The study used stratified whole-group sampling from 6 middle schools and 6 high schools, with three classes selected from each grade, totaling 108 teaching classes. However, the analysis treats all students as independent observations. Because students are nested within classes and schools, standard errors and P-values may be biased if clustering is ignored. The authors should consider multilevel modeling, generalized estimating equations, or cluster-robust standard errors. At minimum, they should justify why clustering was not considered.
Revised response: We appreciate the reviewers’ valuable suggestions and have made the requested revisions.The method section states that participants were selected at random.

5. The manuscript defines ABSI as waist circumference divided by BMI^(2/3) × height^(1/2). HowePFI was calculated using Z-scores based on the 2014 China National Student Physical Fitness Survey, including grip strength, sit-and-reach, standing long jump, 50-m race, and endurance run. However, the manuscript does not provide the national reference means and standard deviations used for each age-sex group.ver, the units and calculation procedure are not sufficiently clear.
Revised response: We appreciate the reviewers’ valuable suggestions and have made the requested revisions. Both the national average reference value in China and the national standard deviation in China are based on data from the 2014 China National Student Physical Fitness Survey. Because the reference data is broken down by age group and gender, there is too much information to include in the manuscript. We have included a list of references in the manuscript. （CNSSCH Association. Report on the 2014th National Survey on Students’Constitution and Health. Beijing: China College & University Press; 2016.）

6. The manuscript should consistently use “a body shape index” rather than “a body size index,”.
Revised response: We appreciate the reviewers’ valuable suggestions and have made the requested revisions.

7. Table 3 is too dense and difficult to read. The authors should simplify the table, move post-hoc pairwise comparisons to supplementary materials, and clearly state the correction method used for multiple comparisons.
Revised response: We appreciate the reviewers’ valuable suggestions and have made the requested revisions. Table 3 has been simplified as requested.

8. Figure 3 shows extreme predicted PFI values at higher ABSI levels, especially in boys, which may reflect extrapolation beyond the observed data range. The authors should show the observed data distribution or restrict fitted curves to the observed ABSI range.
Revised response: We appreciate the reviewers’ valuable suggestions and have made the requested revisions.

Reviewers' comments:

Reviewer's Responses to Questions

**Comments to the Author**

1. Is the manuscript technically sound, and do the data support the conclusions?

The manuscript must describe a technically sound piece of scientific research with data that supports the conclusions. Experiments must have been conducted rigorously, with appropriate controls, replication, and sample sizes. The conclusions must be drawn appropriately based on the data presented.

Reviewer #1: Yes

Reviewer #2: Yes

2. Has the statistical analysis been performed appropriately and rigorously?

Reviewer #1: Yes

Reviewer #2: Yes

3. Have the authors made all data underlying the findings in their manuscript fully available?

The [PLOS Data policy](http://track.editorialmanager.com/CL0/http://www.plosone.org/static/policies.action%23sharing/1/010f019e672f1405-21466ac6-3d9b-4334-a3dd-88379a09ebbe-000000/Wo47epLz8dnDtq20suiAVkKes482PcCphijM-Wzclcc=258) requires authors to make all data underlying the findings described in their manuscript fully available without restriction, with rare exception (please refer to the Data Availability Statement in the manuscript PDF file). The data should be provided as part of the manuscript or its supporting information, or deposited to a public repository. For example, in addition to summary statistics, the data points behind means, medians and variance measures should be available. If there are restrictions on publicly sharing data—e.g. participant privacy or use of data from a third party—those must be specified.

Reviewer #1: Yes

Reviewer #2: Yes

4. Is the manuscript presented in an intelligible fashion and written in standard English?

PLOS ONE does not copyedit accepted manuscripts, so the language in submitted articles must be clear, correct, and unambiguous. Any typographical or grammatical errors should be corrected at revision, so please note any specific errors here.

Reviewer #1: Yes

Reviewer #2: Yes

5. Review Comments to the Author

Please use the space provided to explain your answers to the questions above. You may also include additional comments for the author, including concerns about dual publication, research ethics, or publication ethics. (Please upload your review as an attachment if it exceeds 20,000 characters)

Reviewer #1: the manuscript technically sound,statistical analysis has been performed appropriately,the data is clear but the way of collection needs to be provided ,and the manuscript presented is written in standard English

Revised response: We appreciate the reviewers’ valuable suggestions.

Reviewer #2: This is a commendable piece of research that tackles an important and contemporary issue in public health and physical education. The authors have gathered a compelling dataset, and the manuscript offers a meaningful contribution to the field. To elevate the quality of the paper to its full potential, I have raised a few specific queries regarding the methodology and operational definitions below.

Revised response: We appreciate the reviewers’ valuable suggestions.

Point 84. Reviewer Comment: Could the authors clarify whether validated methods or standardized criteria were employed to establish the selected study population? Please provide the relevant literature or bibliographical references supporting the sampling/selection strategy used.
Revised response: We appreciate the reviewers’ valuable suggestions and have made the requested revisions. “Participants in this study will be evaluated from September to October 2024. Based on the sampling methods used in the China National Student Physical Fitness Survey(27). ”

Point 87. Reviewer Comment: For the sake of clarity and reproducibility, please provide a formal and explicitly cited definition of "chronic disease" as applied within the context of this study.

Revised response: We appreciate the reviewers’ valuable suggestions and have made the requested revisions. “Subjects were included if they had no chronic diseases such as congenital disabilities or heart disease; parents gave informed consent and students voluntarily accepted the survey. In this study, chronic diseases refer to a category of noncommunicable diseases characterized by a long course of illness, gradual onset, complex etiology, and difficulty in achieving a complete cure, requiring long-term monitoring and comprehensive management; examples include hypertension, coronary heart disease, and diabetes. ”

Point 90. Reviewer Comment: It is noted that certain questionnaires were deemed "valid." Could the authors explicitly define the exact inclusion and exclusion criteria used to evaluate the validity or completeness of the returned questionnaires?
Revised response: We appreciate the reviewers’ valuable suggestions and have made the requested revisions. “Subjects were included if they had no chronic diseases such as congenital disabilities or heart disease; parents gave informed consent and students voluntarily accepted the survey. In this study, chronic diseases refer to a category of noncommunicable diseases characterized by a long course of illness, gradual onset, complex etiology, and difficulty in achieving a complete cure, requiring long-term monitoring and comprehensive management; examples include hypertension, coronary heart disease, and diabetes. 104 students were dropped due to incomplete test data. These primarily include key demographic trends, such as age and gender; and incomplete information due to damaged questionnaires. ”

Point 106. Reviewer Comment: Did the study protocol include a follow-up assessment to evaluate the recovery rate or time course of participants following the physical fitness tests? If so, please specify the timeline and parameters measured; if not, consider discussing this aspect as a potential direction for future research or as a limitation.
Revised response: We appreciate the reviewers’ valuable suggestions and have made the requested revisions. “In addition, future studies should evaluate the participants’ post-test status, including their recovery following the physical fitness tests.”

Point 111. Reviewer Comment: To improve the international readability of the manuscript, it would be highly beneficial to explicitly detail the specific methodologies and protocols utilized by the China
National Student Physical Fitness Survey. Providing a brief description or an appropriate reference would significantly help readers unfamiliar with this specific national framework.

I would like to thank the authors for their hard work and for submitting this research.

Revised response: We appreciate the reviewers’ valuable suggestions and have made the requested revisions. Additions have been made to the Methods section of the manuscript.
